# Expert agreement in prior elicitation and its effects on Bayesian inference

**DOI:** 10.3758/s13423-022-02074-4

**Published:** 2022-04-04

**Authors:** Angelika M. Stefan, Dimitris Katsimpokis, Quentin F. Gronau, Eric-Jan Wagenmakers

**Affiliations:** 1 Department of Psychology, University of Amsterdam, Amsterdam, The Netherlands; 2 Department of Psychology, University of Basel, Basel, Switzerland

**Keywords:** Bayes factor, Prior distribution, Hypothesis testing, *t*-Test, Correlation, Robustness, Sensitivity analysis

## Abstract

Bayesian inference requires the specification of prior distributions that quantify the pre-data uncertainty about parameter values. One way to specify prior distributions is through prior elicitation, an interview method guiding field experts through the process of expressing their knowledge in the form of a probability distribution. However, prior distributions elicited from experts can be subject to idiosyncrasies of experts and elicitation procedures, raising the spectre of subjectivity and prejudice. Here, we investigate the effect of interpersonal variation in elicited prior distributions on the Bayes factor hypothesis test. We elicited prior distributions from six academic experts with a background in different fields of psychology and applied the elicited prior distributions as well as commonly used default priors in a re-analysis of 1710 studies in psychology. The degree to which the Bayes factors vary as a function of the different prior distributions is quantified by three measures of concordance of evidence: We assess whether the prior distributions change the Bayes factor direction, whether they cause a switch in the category of evidence strength, and how much influence they have on the value of the Bayes factor. Our results show that although the Bayes factor is sensitive to changes in the prior distribution, these changes do not necessarily affect the qualitative conclusions of a hypothesis test. We hope that these results help researchers gauge the influence of interpersonal variation in elicited prior distributions in future psychological studies. Additionally, our sensitivity analyses can be used as a template for Bayesian robustness analyses that involve prior elicitation from multiple experts.

The past two decades have seen a rise in the popularity of Bayesian methods for data analysis (Andrews & Baguley, 2013). Pragmatic benefits of a Bayesian analysis include the ability to quantify evidence for both the null and the alternative hypothesis, and the ability to monitor the evidence continually as data accumulate (e.g., Wagenmakers et al., 2016, 2018). Bayesian methods also allow researchers to analyze data irrespective of the intention with which these were collected, and yield results that have an intuitive interpretation (Smith, 1965; Gigerenzer, 2004; Ly et al., 2020). By reducing or eliminating the computational and mathematical barriers, software programs such as *JASP* (JASP Team, 2020) and *Stan* (Carpenter et al., 2017; Gelman et al., 2014) have further supported the broad adoption of Bayesian methods.

A core component of the Bayesian statistical framework are prior distributions, that is, probability distributions placed on parameters in Bayesian models. The shape of a prior distribution represents the knowledge about a parameter before data collection. Specifically, peaked distributions that concentrate most mass on a small range of parameter values indicate a high amount of prior certainty, whereas wide distributions that spread their mass across a large range of parameter values indicate a low amount of prior certainty (Dienes, 2008). Information to be incorporated in the prior distribution can be obtained from practical or theoretical considerations, can be derived from earlier studies (e.g., in the case of replication studies), or can be elicited from domain experts (Dienes, 2019; Ly et al., 2019; O’Hagan et al., 2006). It has been shown repeatedly that the results of Bayesian analyses, and especially Bayes factor hypothesis testing, can be sensitive to the specification of the prior distribution. Researchers should therefore dedicate special attention to the specification of prior distributions in the model development process (e.g., Berger, 1990; Sinharay & Stern, 2002).

A frequently voiced concern is that the shape of informed prior distributions is to some extent arbitrary because it relies on the subjective opinions of single researchers or field experts, poorly justified decisions in the prior elicitation procedure, or on the idiosyncrasies of previous studies (Depaoli & van de Schoot, 2017; Stefan et al., 2020). Practitioners who do not wish to jeopardize the objectivity of their statistical analyses are therefore often reluctant to incorporate a high level of prior information into the prior distribution. Instead, they may prefer default prior distributions that satisfy certain mathematical desiderata and display a high amount of uncertainty about parameter values (Jaynes, 1968; Kass & Raftery, 1995; Lee & Vanpaemel, 2017; Consonni et al., 2018; Bayarri et al., 2012a). However, as they are not designed for any particular application domain, default prior distributions ignore relevant theoretical, practical, and empirical information. For example, default prior distributions do not incorporate theoretically motivated constraints on parameter values (Vanpaemel & Lee, 2012), knowledge about common empirical parameter values from earlier studies (Matzke & Wagenmakers, 2009; Tran et al., 2020), or knowledge about practical constraints arising from a specific study design (Dienes, 2019). Therefore, they run the risk of leading to unrealistic model predictions, and may decrease the diagnosticity of Bayesian model comparisons (Lee & Vanpaemel, 2017; Stefan et al., 2019). Thus, despite being potentially more susceptible to interpersonal variation, informed prior distributions have important theoretical advantages over default prior distributions.

One method to specify informed prior distributions is through prior elicitation from experts (O’Hagan et al., 2006; Dias et al., 2018; Mikkola et al., 2021). Prior elicitation can be described as an interview procedure where a researcher guides one or more field experts through the process of expressing their domain knowledge in a probabilistic form (Winkler, 1967; Garthwaite et al., 2005). The participating field experts can be researchers themselves or practitioners who possess relevant empirical insights, such as psychotherapists, doctors, or teachers (Thall & Cook, 2004; Bolsinova et al., 2017; Mossman et al., 2015; Gronau et al., 2020). Within the past 50 years, a multitude of prior elicitation methods have been proposed that range from highly model-specific to broadly applicable standard methods (for overviews, sees Garthwaite et al.,, 2005; Johnson et al.,, 2010; Grigore et al.,, 2013). A key objective of prior elicitation methods is to minimize the cognitive biases that can emerge in probability assessments (O’Hagan, 2019; Kahneman, 2011). Therefore, several popular elicitation methods apply an indirect approach where experts are not asked to provide probability statements directly, but are instead asked to bet on parameter values (Johnson et al., 2010) or to assess the plausibility of future data (Winkler, 1967; Kadane, 1980).

Prior distributions obtained from an elicitation effort are particularly open to concerns of subjectivity. The results of a prior elicitation procedure crucially depend on the participating experts and their views of the research problem at hand. Therefore, a common recommendation is to elicit priors from multiple experts with different backgrounds to explore the interpersonal variability of elicitation results (Aspinall, 2010; Grigore et al., 2013; Chaloner, 1996). However, this advice is rarely heeded in practice. Often enough, priors in psychological research are elicited from single experts (e.g., Gronau et al.,, 2020) or directly combined into a single aggregated prior distribution that incorporates information from all experts (e.g., Bolsinova et al.,, 2017; Mossman et al.,, 2015). The variability of elicited prior distributions and its effect on the results of Bayesian inference are rarely studied explicitly (but see, e.g., Veen et al.,, 2018). We argue that this makes it difficult for substantive researchers to gauge the effect of the interpersonal variability of elicitation results on Bayesian inference, which in turn may increase the discomfort that researchers feel concerning the use of prior elicitation methods in their own research.

In this article, we demonstrate the effects of interpersonal variability in prior distributions using elicited priors from six experts with a background in different fields of psychology. Specifically, we investigate how the differences between the elicited prior distributions affect Bayes factor null hypothesis testing. We focus on Bayes factors because, unlike for posterior distributions (Wrinch & Jeffreys, 1921), the influence of the prior on the Bayes factor does not become negligible with large amounts of data. To analyze the influence of the prior distributions, we calculate Bayes factors for 855 *t*-tests and 855 correlation tests extracted from psychological literature (Wetzels et al., 2011; Bosco et al., 2015), and provide measures for the sensitivity of the Bayes factor to the choice of the prior distributions for these tests. We believe that making the variability of results explicit for a large number of independent psychological data sets will help researchers gauge the influence of expert selection in future psychological studies. Additionally, our sensitivity analyses can be used as a template for prior sensitivity analyses in the future, where prior distributions are elicited from several experts.

Our article is structured as follows. First, we describe the method we used to elicit the prior distributions. Then, we present the prior distributions that resulted from our elicitation effort, and discuss their interpersonal differences. Next, we reanalyze correlation tests and *t*-tests obtained from two large meta-analytical databases spanning multiple psychological disciplines (Wetzels et al., 2011; Bosco et al., 2015). In the Bayes factor hypothesis tests, we use the different elicited prior distributions as well as default priors that are common standards for the respective hypothesis tests. Our sensitivity analysis focuses on three questions: (1) “How often do the priors change the direction of the Bayes factor?”, targeting the issue that different priors can lead to support for different hypotheses, (2) “How often do the priors change the evidence category?”, targeting the issue that different priors can lead to support that falls into different categories of evidence strength, and (3) “How much do the priors change the value of the Bayes factor?”, targeting the issue that different priors lead to quantitative differences between Bayes factors. We believe these three questions cover the central aspects that determine the conclusions that researchers draw about hypotheses based on a Bayes factor hypothesis test, which makes these questions an important target for sensitivity analyses.

## Elicitation method

Six post-doctoral researchers and professors from the University of Amsterdam participated in the study: Two social psychologists, two cognitive neuroscientists, and two developmental psychologists. The participants were contacted a few days before the interview and agreed to participate in the study on the basis of a brief description of the procedure. They did not receive any monetary compensation for their participation.

The elicitation setup emulated a typical situation in psychological research where a directional alternative hypothesis is tested and small-to-medium sized effect sizes can be expected. The elicitation procedure took place in the form of a semi-structured face-to-face interview.

At the beginning of the interview, participants were informed that the goal of the elicitation task was to assess their expectations for small-to-medium effect sizes in their respective field of study. This deviates from a standard prior elicitation procedure insofar as that typically, experts would be queried about their expectations for specific effects in their field of study (e.g., the Facial Feedback effect in social psychology, see Gronau et al. 2020), and potentially even about a specific experimental design (Dienes, 2019). Here, we decided for a more general elicitation target as this allowed us to uncouple our elicitation procedure from an idiosyncratic research context and establish a minimum level of consent between experts (i.e., experts agree on the existence and direction of the effect, and would use the same label to describe its size). A minimum level of consent between experts can be regarded as desirable, as it is unclear whether experts are capable of formulating unbiased predictions for a theoretical scenario that disagrees with their convictions (Stefan et al., 2020).

Subsequently, Cohen’s *δ* and the Pearson correlation coefficient *ρ* (Cohen, 1988, 1992) were introduced as examples for effect size measures in the context of the comparison of means and correlation tests, respectively. Participants were further informed that the purpose of the elicitation procedure was to assess their expectations for the case that a one-sided alternative hypothesis is true, that is, a scenario where the effect size is larger than zero. They were told that they would be able to change their assessments at any time during the course of the interview.

**Fig. 1:**
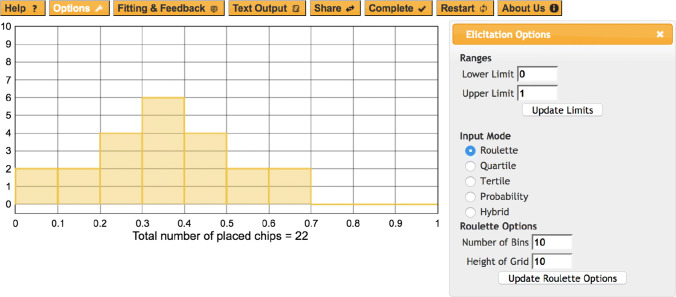
Example for eliciting a prior distribution using the Roulette method in the MATCH tool (Morris et al., 2014)

**Fig. 2:**
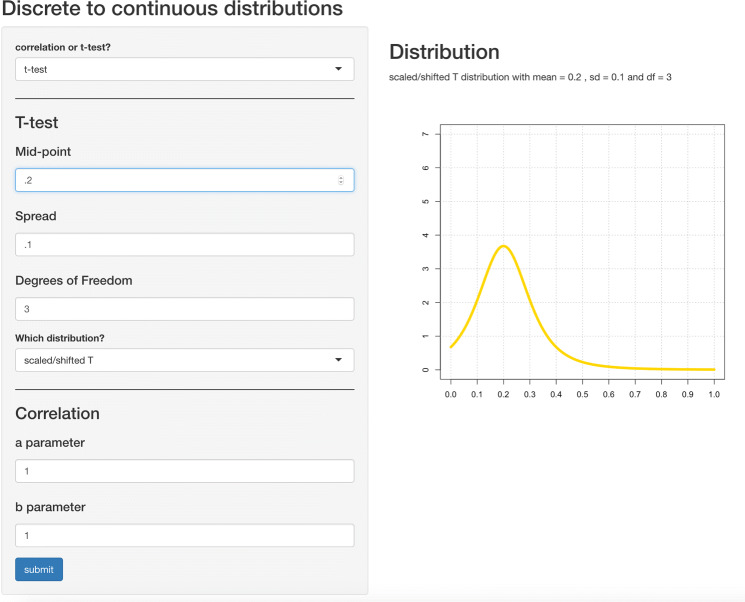
Example for adjusting an elicited distribution using the custom-made Shiny app

The prior elicitation followed the Histogram Method, where experts communicate their subjective prior distribution by using the bars of a histogram (van Noortwijk et al., 1992). The Histogram Method is one of the most frequently used elicitation approaches and is claimed to be accessible to experts regardless of their level of statistical knowledge (Grigore et al., 2013; Bolger, 2018). We used the MATCH software (Morris et al., 2014) in combination with a custom-made Shiny app to support the elicitation procedure. A screenshot of the MATCH tool and of the Shiny app can be found in Figs. 1 and 2, respectively. At the beginning of the Histogram Method, the participants were asked two questions: (1) “Imagine how general small-to-medium effect sizes in your field would look like. Which effect size would you expect as the most probable one to be found?”, (2) “Which range of values would you consider possible?”. Subsequently, the expert was asked to place virtual chips on the MATCH elicitation grid in a way that reflects their assessment of the plausibility of the values in the grid. The more plausible an expert regards a certain range of values, the more chips they place on that range. The grid consisted of ten bins of effect size values ranging from 0 to 1, and a maximum of ten chips could be placed in each bin. Participants were given as much time as they needed to place the chips, and could at any time turn to the interviewer in case of questions.

After participants had placed their chips, the fitting procedure in the MATCH tool was used to fit a probability distribution to the results of the elicitation. Following this step, the fitted parameters of the distribution were transferred to a Shiny app (see Fig. 2) where participants were able to adjust the parameters of the distribution, if they felt that the fitted distribution did not perfectly represent their prior beliefs. At participants’ request, a brief explanation of the meaning of each parameter was provided (e.g., that the standard deviation specifies the spread of the distribution).

The process was repeated separately for Cohen’s *δ* and the Pearson correlation coefficient. For the correlation coefficient, the elicited prior distribution took the form of a beta-distribution with parameters *α* and β. For Cohen’s *δ*, participants were asked to adjust a fitted normal distribution as well as a fitted scaled and shifted *t*-distribution because we expected them to be more familiar with the parameters of the normal distribution, but wanted to provide them with the added flexibility of the flatter tails of a *t*-distribution. Since the elicited normal and *t*-priors differed only marginally, we will only report our results for the *t*-priors in the following. Results for the elicited normal priors can be found in the Online Appendix (https://osf.io/vqszj/).

## Elicited prior distributions

**Fig. 3:**
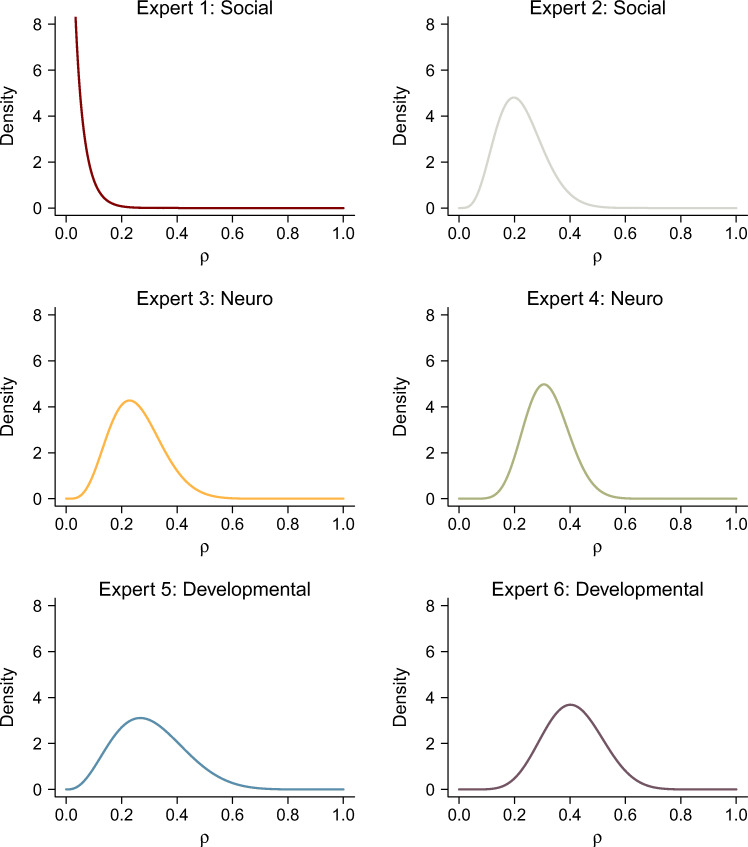
Elicited prior distributions for the Pearson correlation coefficient *ρ* for all six experts. The colors of the experts’ distributions match the colors used in later figures

**Fig. 4:**
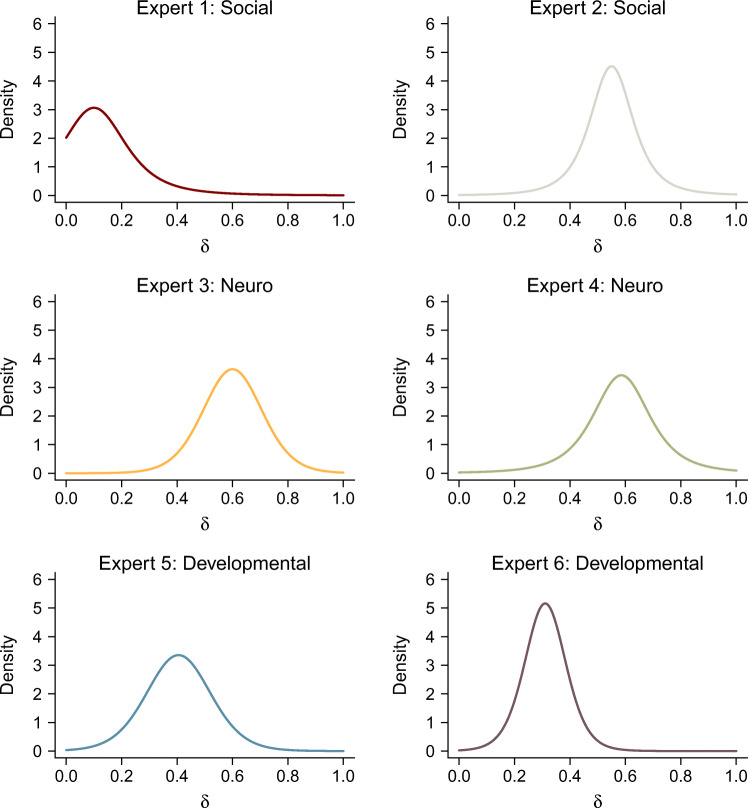
Elicited *t*-distribution priors for Cohen’s *δ* for all six experts. The colors of the experts’ distributions match the colors used in later figures

The elicited prior distributions are shown in Fig. 3 and 4, respectively, and the parameters of the elicited distributions can be found in Table 1.

**Table 1:** Elicited parameters of the beta and *t*-distribution priors

		Elicitated priors				
		Beta prior		t-prior		
Expert	Field	*α*	β	*μ*	*σ*	*ν*
1	Social Psychology	0.62	22.44	0.10	0.12	3
2	Social Psychology	5.32	18.58	0.55	0.08	3
3	Cognitive Neuroscience	5.35	15.69	0.60	0.11	13
4	Cognitive Neuroscience	10.70	22.98	0.59	0.11	3
5	Developmental Psychology	3.83	8.76	0.41	0.12	13
6	Developmental Psychology	8.65	12.39	0.31	0.08	9

The Greek letters stand for each parameter: *α* and β for the alpha and beta parameters of the beta-distribution; *μ* for the mean, *σ* for the scale parameter, and *ν* for the degrees of freedom of the *t*-distribution

For the Pearson correlation coefficient, all experts placed most prior distribution mass on values smaller than *ρ* = 0.5. Expert 1 differs markedly from the other experts by assigning a high probability to correlation coefficients close to zero. Expert 6 made the most optimistic claims about the correlation coefficient by placing the peak of their distribution on values around a correlation coefficient of *ρ* = 0.4. The elicited priors of all other experts are relatively similar with peaks around values between 0.2 and 0.3. Compared to the other experts, Expert 5 has a somewhat wider prior distribution that signifies more uncertainty about the size of a small-to-medium effect size in their field. Note that the assessments of Experts 2–5 are roughly in agreement with Cohen’s (1988, pp. 79f.) classification scheme, according to which Pearson correlation coefficients between *ρ* = 0.1 and *ρ* = 0.3 reflect small-to-medium-sized effects.

For Cohen’s *δ*, experts differed to a similar degree in their elicited prior distributions. The peaks of the prior distributions ranged from *δ* = 0.1 (Expert 1) to *δ* = 0.6 (Expert 3). Consistent with the elicited priors for the correlation coefficient, Expert 1 expected substantially lower effect sizes than the other experts. Expert 6 showed the least uncertainty about the parameter (i.e., the most peaked prior distribution), with 95% of the distribution between *δ* = 0.14 and *δ* = 0.48. There was, again, considerable consistency between the elicited prior distributions of Experts 2–5. For Cohen’s *δ*, several experts’ prior distributions did not match Cohen’s classification of a small-to-medium effect size. Cohen classified *δ* = 0.2 as a small, and *δ* = 0.5 as a medium effect size (Cohen, 1988, pp. 25f.), whereas several experts placed considerable weight on effect sizes larger than *δ* = 0.5. For example, Experts 2, 3, and 4 all placed more than 70% of their prior distribution on values larger than *δ* = 0.5. Therefore, most experts considered small-to-medium effects to be larger than their normative definition.

**Fig. 5:**
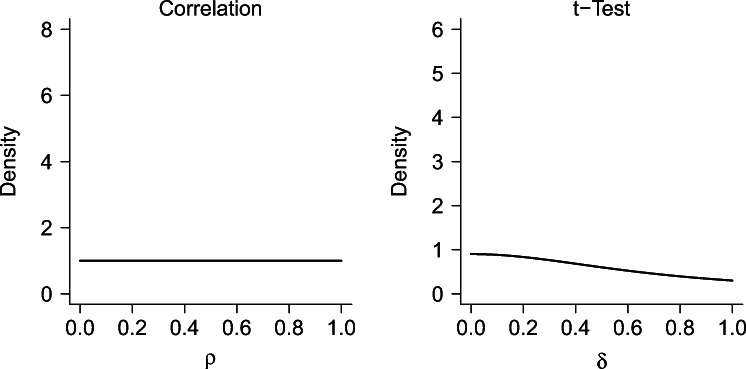
Default prior distributions for the one-sided Bayesian correlation test and *t*-test. The default prior for the correlation test is a uniform distribution from 0 to 1. The default one-sided prior for the *t*-test is a positive-only Cauchy distribution with mode 0 and scale parameter $\sqrt {2}/2$ (mass greater than *δ* = 1 not shown here)

As can be expected, all elicited prior distributions differ substantially from the default prior distributions commonly used for Bayesian *t*-tests and correlation tests (see Fig. 5). The elicited prior distributions reflect less uncertainty about parameter values and—apart from Expert 1—none of the experts assigned considerable prior mass to parameter values close to zero. Therefore, with the possible exception of Expert 1, the elicited prior distributions can be said to be more similar to one another than to the default prior distribution.

## Reanalyzing hypothesis tests from the psychological literature

In the following sections, we will reanalyze hypothesis tests extracted from the psychological literature using the elicited prior distributions. The goal is to showcase the extent to which the differences between elicited prior distributions can influence the results of Bayesian hypothesis testing. We will apply the elicited beta-distribution priors and *t*-distribution priors to correlation tests and *t*-tests, respectively. We will compare the results among the elicited prior distributions as well as with the results of hypothesis tests using default prior distributions. The code to reproduce the results can be found in the online supplementary materials (https://osf.io/vqszj/).

### The Bayesian hypothesis testing procedure

In our comparisons, we will focus on Bayes factors as the central outcome of the Bayesian hypothesis test. The Bayes factor is a measure of relative evidence provided by the data for one model over another (Kass & Raftery, 1995). For example, a Bayes factor of BF_10_ = 6 means that the data are six times more likely under the alternative hypothesis (${\mathscr{H}}_{1}$) than under the null hypothesis (${\mathscr{H}}_{0}$). Bayes factors larger than 1 can be interpreted as evidence in favor of the alternative hypothesis, while Bayes factors smaller than 1 can be interpreted as evidence in favor of the null. Mathematically, the Bayes factor is defined as a ratio of two prior-weighted averaged likelihoods,

1 $$ \text{BF}_{10} = \frac{p(\mathcal{D} \mid \mathcal{H}_{1})}{p(\mathcal{D} \mid \mathcal{H}_{0})} = \frac{\int p(\mathcal{D} \mid \theta_{1}, \mathcal{H}_{1}) p(\theta_{1} \mid \mathcal{H}_{1})\text{d}\theta_{1}}{\int p(\mathcal{D} \mid \theta_{0}, \mathcal{H}_{0}) p(\theta_{0} \mid \mathcal{H}_{0})\text{d}\theta_{0}}  , $$

where $p(\theta _{1} \mid {\mathscr{H}}_{1})$ and $p(\theta _{0} \mid {\mathscr{H}}_{0})$ are the prior distributions under the alternative and null model, and $p(\mathcal {D} \mid \theta _{1}, {\mathscr{H}}_{1})$ and $p(\mathcal {D} \mid \theta _{0}, {\mathscr{H}}_{0})$ are the likelihood functions under the alternative and null model, respectively. In Bayesian null hypothesis testing, under ${\mathscr{H}}_{0}$, the parameter of interest (e.g., effect size) is typically assigned a point prior that puts all mass on a null value (*ρ* = 0 and *δ* = 0 in our case); for the nuisance parameters (e.g., the variance) wide default prior distributions are specified (Ly et al., 2016).^1^ The null hypothesis therefore represents the idealized position of a sceptic.

In contrast, under ${\mathscr{H}}_{1}$, the parameter of interest is assumed to be different from zero, and the uncertainty about its true value is reflected in a prior distribution. These prior distributions can either be elicited, as presented above, or they can be specified as defaults designed to meet particular desiderata (e.g., Bayarri et al. 2012b). As a default prior distribution for the correlation test, we use a uniform distribution on the correlation coefficient *ρ*, as recommended by Jeffreys (1961, pp. 174–179 and 289–292, see also Ly et al. 2018). For the *t*-test, our default distribution is a central Cauchy distribution with a scale parameter of $\sqrt 2/2$ on effect size Cohen’s *δ*, as recommended by Morey and Rouder (2018). Both default prior settings are also implemented in *JASP* (JASP Team, 2020). Figure 5 displays the default prior distributions. For consistency with our elicitation procedure, the default prior on *δ* is positive-only, that is, we assume that the hypothesized direction for the effect is known.

### Meta-analytic databases

**Fig. 6:**
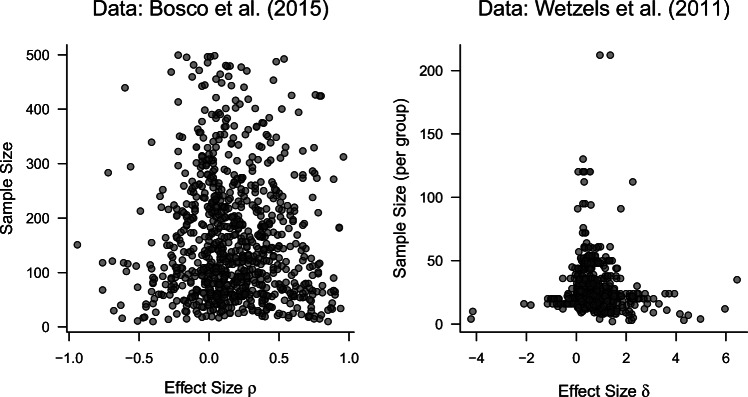
Distribution of effect sizes and sample sizes in the two meta-analytic databases used in this paper (Bosco et al., 2015; Wetzels et al., 2011)

We reanalyze hypothesis tests from two large psychological databases. For the *t*-test, we compute Bayes factors for the meta-analytic database assembled by Wetzels et al., (2011). The database contains a total of 855 *t*-tests reported in 252 articles from the 2007 issues of *Psychonomic Bulletin & Review* and the *Journal of Experimental Psychology: Learning, Memory, and Cognition*. The *t*-tests include 85 one-sample *t*-tests, 604 paired samples *t*-tests, and 166 independent samples *t*-tests, with sample sizes ranging from 2 to 212 (per group), and a median sample size of 24. The sample effect sizes for *δ* range from *d* = − 4.23 to *d* = 6.44, with a median of *d* = 0.57. The distribution of effect sizes in the Wetzels et al. database can be seen in the right panel of Fig. 6.

For the correlation tests, we reanalyze data from a database assembled by Bosco et al., (2015). The latest version of the database (version 2.08, see http://www.frankbosco.com/data/CorrelationalEffectSizeBenchmarks.html) contains a total of 172,492 correlation coefficients extracted from journal articles in *Personnel Psychology* and the *Journal of Applied Psychology* between the years 1980 and 2010. For practical reasons, we use a random subset of 855 correlation coefficients from this database. These coefficients were extracted based on the following rules: First, we removed all perfect correlations (*r* ∈{− 1,1}) since these do not typically represent psychologically meaningful relationships. Then, we removed all correlation coefficients for which the database indicated unequal sample sizes or non-integer sample sizes for the two measured variables. In a third step, we removed correlations based on sample sizes smaller than ten and larger than 500 for computational purposes. From the remaining data, we sampled 855 correlation coefficients, matching the number of coefficients reported in the Wetzels et al., (2011) database. Correlation coefficients were sampled from different studies to ensure independence between the correlation coefficients. The final set of correlation coefficients ranges from *r* = − 0.94 to *r* = 0.96, with a median correlation of *r* = 0.15. The distribution of correlation coefficients in the Bosco et al. database can be seen in the left panel of Fig. 6.

### Question 1: How often do the priors change the direction of the Bayes factor?

For many researchers, a key outcome of a Bayesian hypothesis test is the direction of the Bayes factor: Do the data support the null hypothesis or do they support the alternative hypothesis? Even though the mere direction of the Bayes factor should be interpreted with care, especially if the evidence is only weak, the direction of the Bayes factor is generally of great importance when interpreting the results of an experiment (Jeffreys, 1938, pp. 377–378). Therefore, our first sensitivity analysis concerns the direction of the Bayes factor. If the direction of the Bayes factor remains the same, regardless of the prior distribution used, the main conclusion of the hypothesis test is robust against the choice of the prior.

**Fig. 7:**
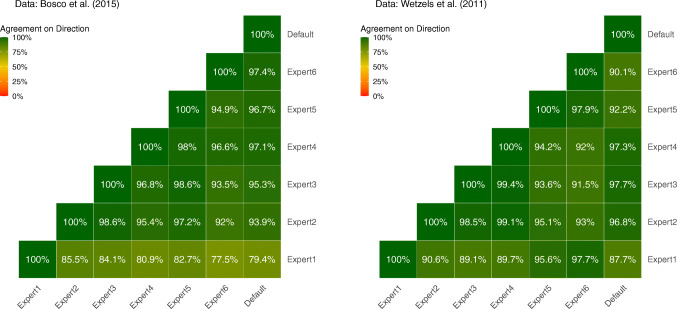
Agreement rates of Bayes factors with regard to the direction of evidence for all combinations of prior distributions. Agreement criterion: Both Bayes factors are either larger than 1 or smaller than 1

Figure 7 shows how often the Bayes factors computed for the different elicited prior distributions point in the same direction. We defined the agreement rate as the proportion of tests where both Bayes factors are either larger or smaller than BF_10_ = 1. Generally, there is a high agreement between the Bayes factors for our elicited priors. For most combinations of prior distributions, the Bayes factor points towards the same hypothesis in over 90% of the conducted tests. The largest influence of the prior distribution can be observed for the prior distribution of Expert 1. Here, agreement with the other Bayes factors goes down to a minimum of 77.5% for the Bosco et al., (2015) data and 87.7% for the Wetzels et al., (2011) data. As is evident from Figs. 3 and 5, the elicited prior distributions for Expert 1 differ substantially from those of the other experts, primarily because Expert 1 assigned a relatively large proportion of prior mass to values near zero.

Notably, for our sample of expert-elicited prior distributions, most of the time, Bayes factors using the default prior distribution pointed to the same direction as Bayes factors based on our elicited priors. Even Expert 1 reached agreement rates of 79.4% or higher with the default prior. This indicates that for psychological data, elicited prior distributions need to differ substantially from the default prior to change the direction of the result of the hypothesis test.

### Question 2: How often do the priors change the evidence category?

An important goal of a Bayesian hypothesis test is to measure the strength of evidence in favor of the null hypothesis versus the alternative hypothesis. The Bayes factor allows for a continuous quantification of the strength of evidence in favor of either hypothesis. However, in interpreting the Bayes factor, researchers often rely on rough heuristic classifications of evidence strength. For example, according to Jeffreys’ (1961) classification, Bayes factors between 1 and 3 can be categorized as anecdotal evidence, Bayes factors between 3 and 10 indicate moderate evidence, and Bayes factors above 10 are labeled as strong evidence. Even though all evidence classification systems are arbitrary to a certain extent, “jumping” across the thresholds in a particular classification system is often perceived as a qualitative change in the amount of evidence (Tendeiro and Kiers, 2019). In fact, Robinson (2019) pointed out that it is a strength of Bayesian hypothesis tests that their results can fall into either of three categories: Evidence for the null hypothesis, evidence for the alternative hypothesis, or inconclusive evidence. What degree of evidence can be interpreted as convincing evidence depends on the research field (Schönbrodt and Wagenmakers, 2018). For example, a Bayes factor larger than 10 or smaller than 1/10 could be interpreted as convincing evidence in favor of ${\mathscr{H}}_{1}$ or ${\mathscr{H}}_{0}$, respectively, whereas a Bayes factor between these upper and lower bounds might be interpreted as inconclusive evidence. When investigating the sensitivity of the Bayes factor to the prior distribution, it is therefore interesting to evaluate how often a certain evidence threshold has been crossed due to the choice of the prior distribution.

**Fig. 8:**
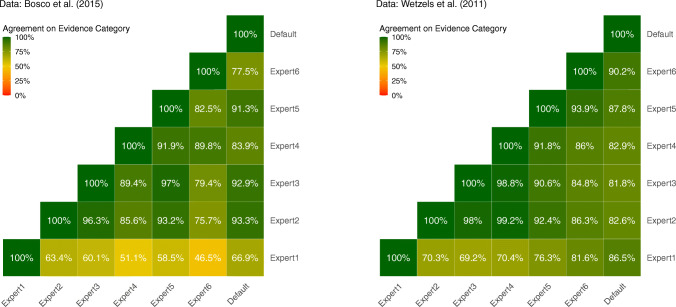
Agreement rates of Bayes factors with regard to the evidence category for all combinations of prior distributions. Here, strong evidence is defined as BF_10_ > 10 or BF_10_ < 1/10. Bayes factors are considered to possess the same strength of evidence if both Bayes factors show strong evidence for the same hypothesis or if both Bayes factors show inconclusive evidence

In Fig. 8, we depict how often Bayes factors crossed an evidence threshold if we applied a different elicited prior distribution or the default prior distribution. As delineated above, we used evidence thresholds of BF_10_ = 10 and BF_10_ = 1/10 to identify strong evidence in favor of ${\mathscr{H}}_{1}$ and ${\mathscr{H}}_{0}$, respectively (for results with other thresholds see our Online Appendix https://osf.io/vqszj/). We recorded a change in the strength of evidence if one of the Bayes factors would be classified as strong evidence while the other Bayes factor would be classified as inconclusive evidence or evidence in favor of the other hypothesis according to these evidence thresholds.

Overall, we can see that the agreement of Bayes factors with regard to the evidence category is lower than the agreement with regard to the direction. Although many Bayes factors agree on the strength of evidence in 90% of the tests or more, several combinations of our elicited prior distributions only yield agreement rates of 80% or less. The agreement rates for Expert 1 are even lower, with rates as low as 47%. This divergence can again be explained by the large difference between Expert 1’s prior distribution and the prior distributions of the other experts. However, with the given data and evidence thresholds, it never occurs that one Bayes factor shows strong evidence in favor of the alternative hypothesis while the matching Bayes factor shows strong evidence in favor of the null hypothesis.

In general, evaluating agreement across two cut-points will result in lower agreement than evaluating agreement across a single cut-point. This provides an intuitive explanation for the lower agreement rates for the strength of evidence compared to the direction of Bayes factors.

### Question 3: How much do the priors change the value of the Bayes factor?

Both the direction and the classification of the Bayes factor are based on a discrete interpretation of the available evidence. Although useful as a rough heuristic, many proponents of Bayesian methods prefer to report the exact value of the Bayes factor, as every discretization leads to a loss of information (e.g., Jeffreys, 1938; van Ravenzwaaij and Wagenmakers, 2019). Below we examine the degree to which the exact values of the Bayes factor change as a result of adopting a different prior distribution.

**Fig. 9:**
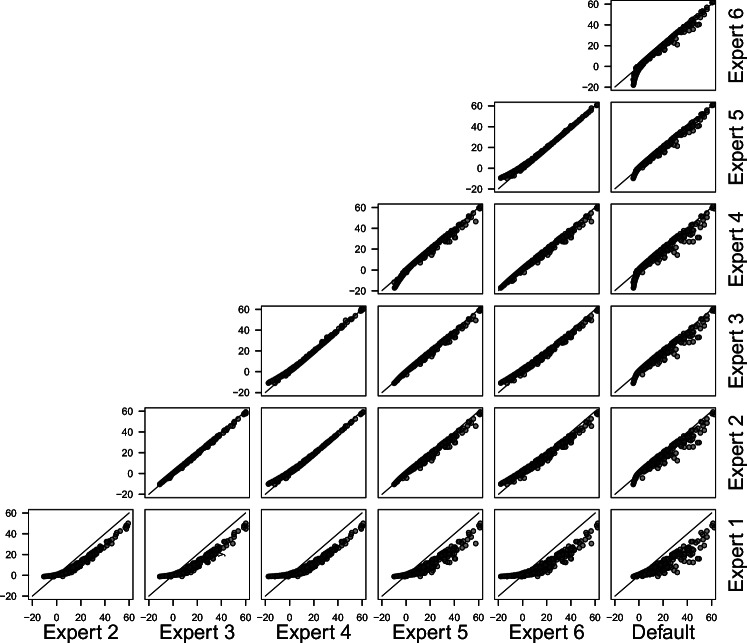
Correspondence between log Bayes factors for all prior distributions in the Bosco et al., (2015) database. The diagonal line marks equal values

**Fig. 10:**
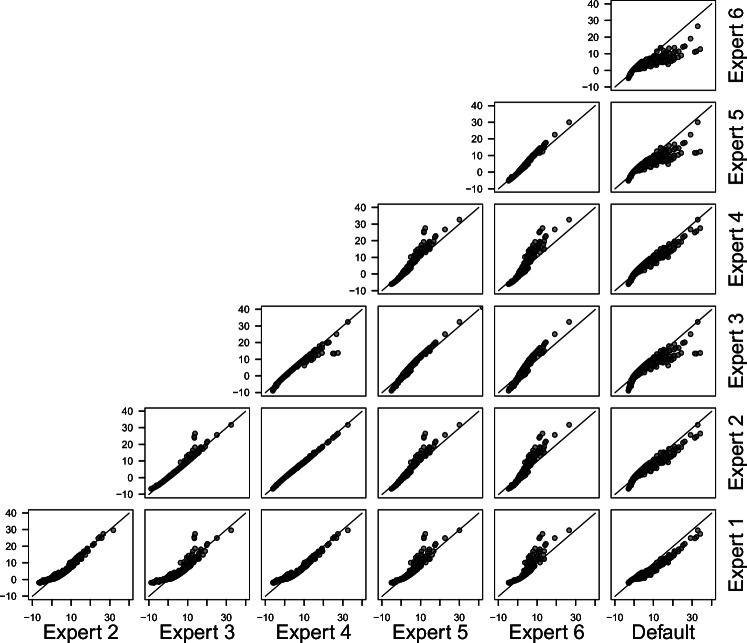
Correspondence between log Bayes factors for all prior distributions in the Wetzels et al., (2011) database. The *diagonal line* marks equal values

Figures 9 and 10 display the correspondence of log Bayes factors for all experts in the two meta-analytic databases. Points falling on the diagonal line signal perfect correspondence, while points falling below or above the line signal higher Bayes factors for the expert plotted on the *x*- or *y*-axis, respectively. We chose to show log Bayes factors because they make it possible to display very large Bayes factors without losing information about smaller Bayes factors. However, it is necessary to keep in mind that due to the logarithmization even small deviations from the diagonal signal large absolute differences in Bayes factors if the Bayes factors are large.^2^ From the figures, it becomes clear that Bayes factors are not always larger or smaller for one prior distribution compared to another, but that the relation differs per study. For example, for some studies, elicited distributions yield larger Bayes factors than the default prior distributions, and for others vice versa.

**Fig. 11:**
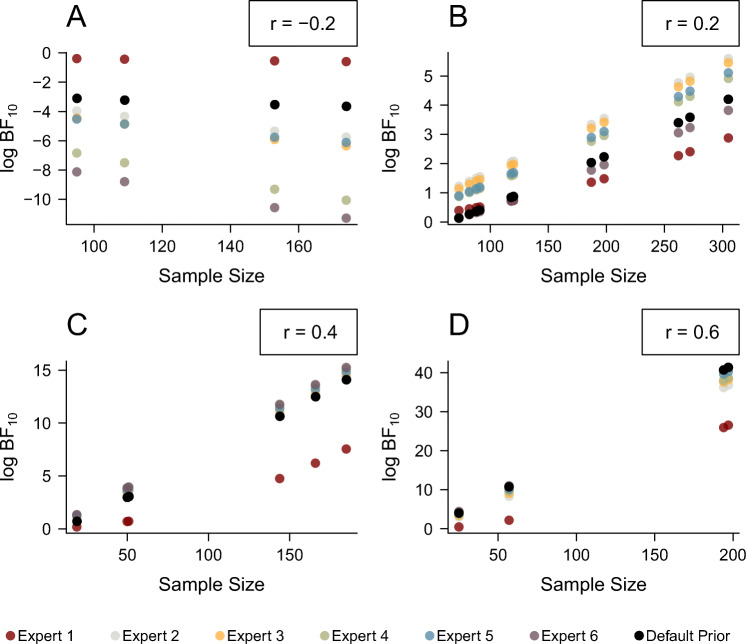
Variation in log Bayes factors for four different observed effect sizes (panels **A**-**D**) in the Bosco et al., (2015) database depending on different priors (color coded) and sample sizes

Figure 11 shows that the effect size in the sample determines which prior distribution yields the highest Bayes factor for a study. The sample size has an additional effect, with larger sample sizes leading to more pronounced differences between the Bayes factors for different prior distributions.

Panel A of Fig. 11 shows log Bayes factors in the Bosco et al., (2015) database for studies with a sample effect size of *r* = − 0.2.^3^ Since a negative sample effect size is inconsistent with the directional alternative hypothesis postulated by the experts, the evidence should point towards the null hypothesis, that is, the log Bayes factors should be negative.^4^ It is easy to see that Expert 1’s prior distribution led to weaker evidence for the null hypothesis than all other prior distributions. This can be explained by the shape of the prior distribution: By placing much weight on effect sizes close to zero, the alternative model of Expert 1 becomes very similar to the null model. Therefore, large sample sizes are necessary to discriminate between the two models. The strongest evidence for the null model is obtained by Expert 6. Expert 6’s prior distribution has a higher mode than all other prior distributions. Negative effect sizes are therefore highly inconsistent with their alternative model and lead to strong support for the null model.

Panels B and C of Fig. 11 show the log Bayes factors for studies in the Bosco et al., (2015) database with sample effect sizes of *r* = 0.2 and *r* = 0.4, respectively. These correlation coefficients were deemed most likely by Expert 2 and Expert 6, respectively, thus yielding a higher predictive accuracy for these experts compared to the other experts and the default prior. The Bayes factor rewards the experts’ predictive accuracy, showing the highest support for the expert’s model who made the best predictions.

Panel D of Fig. 11 shows the log Bayes factors for studies with a sample correlation coefficient of *r* = 0.6 in the Bosco et al., (2015) database. This effect size is larger than the 95th percentile of all elicited prior distributions, which means that none of the experts made accurate predictions. In this case, the default prior distribution gains advantage over the elicited distributions, since it assigned considerable mass to very large effect sizes. However, it is important to note that the prior mass in the default prior distribution is distributed across a wide range of effect sizes. This means that even though the default Bayes factors outscore the informed Bayes factors in our case, an informed prior distribution that predicts large effect sizes instead of small-to-medium effect sizes would lead to even higher Bayes factors than the default distribution. Generally, for large effect sizes, most Bayes factors are highly compelling regardless of the prior that was used because, all else being equal, Bayes factors increase monotonically with increasing effect size.

Our results show that absolute differences between the Bayes factors can be substantial. For instance, for a correlation of *r* = 0.3 and a sample size of 260, Expert 4 has a Bayes factor of 110,157 in favor of the alternative model, while Expert 6 shows evidence of 60,436 in favor of the alternative model. Thus, even for moderate sample sizes, differences in Bayes factors can easily range in the thousands. However, for practical purposes the difference is irrelevant: both Bayes factors display overwhelming evidence in favor of the alternative model. This also becomes clear from the posterior model probability, which is $p({\mathscr{H}}_{1} \mid \mathcal {D}) = 0.999991$ for Expert 4 and $p({\mathscr{H}}_{1} \mid \mathcal {D}) = 0.999984$ for Expert 6 (assuming equal prior model probabilities). It is arguably difficult to picture a scenario in which these differences in posterior model probability would lead to different conclusions or instigate different actions in practice. As stated by Jeffreys,

“We do not need *K* [i.e., BF_01_] with much accuracy. Its importance is that if *K* > 1 the null hypothesis is supported by the observations, while if *K* is very small the null hypothesis may be rejected. But it makes little difference to the null hypothesis whether the odds are 10 to 1 or 100 to 1 against it, and no difference at all whether they are 10^4^ or 10^4000^ to 1; in any case, whatever alternative is most strongly supported will be set up as the hypothesis for use until further notice.” (Jeffreys, 1939, Appendix I, p. 357)

For our sample of elicited priors, it rarely happens that one Bayes factor shows barely any evidence while another Bayes factor shows overwhelming evidence in one direction. Our analyses indicate that, typically, when differences between Bayes factors are large, all Bayes factors are large. This also explains our results in the previous section where we observed a high agreement between the Bayes factors with regard to the evidence category, despite of the large differences between the absolute Bayes factor values.

On a more general account, it should be noted that differences in Bayes factors do not lend themselves to an intuitive interpretation because the Bayes factor lacks a unit of measurement. For example, an absolute difference of 49,721 between the Bayes factor of Expert 4 and Expert 6 might seem large, but cannot be put in perspective unless the values of the Bayes factors involved in the difference are known. In contrast to differences, *ratios* of Bayes factors can be meaningfully interpreted. Due to the principle of transitivity, the ratio between two Bayes factors, BF_10_/BF_20_, is, again, a Bayes factor (BF_12_; Etz et al.,, 2018). For example, the ratio between the Bayes factors of Expert 4 and Expert 6 for a correlation of *r* = 0.3 and a sample size of 260 is 110,157/60,436 = 1.82, meaning that the data are roughly twice as likely under Expert 4’s model than under Expert 6’s model. Thus, even if there is a large absolute difference in Bayes factors, the difference in the quality of prediction for the rival expert models can be small. When interpreting the sensitivity of the Bayes factor to the specification of the prior, it is therefore recommended to analyze the ratios of Bayes factors rather than the absolute value of the Bayes factor difference.

**Fig. 12:**
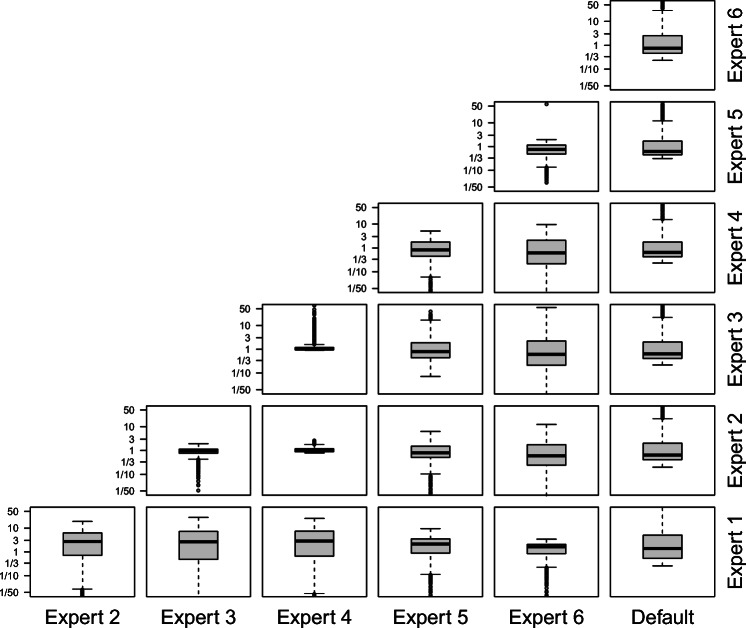
Ratios of Bayes factors of different experts in the Wetzels et al., (2011) database. Ratios are computed by dividing the Bayes factor of the expert on the *x*-axis by the Bayes factor of the expert of the *y*-axis, e.g., the bottom right panel shows the distribution of Bayes factor ratios for BF_Default:Expert1_

Figure 12 shows the distribution of Bayes factor ratios for different experts in the Wetzels et al., (2011) dataset.^5^ From the *y*-axis in each panel, it becomes clear that Bayes factor ratios mostly range between 1/3 and 3, and are rarely smaller than 1/3 or larger than 50, so the predictive accuracy of two expert models is often similar. Note that the information about absolute size of the focal Bayes factor BF_10_ in a hypothesis gets lost when computing the ratio of two Bayes factors, as the marginal likelihood of the null hypothesis cancels out. For sensitivity of hypothesis testing results, Bayes factor ratios for different experts should therefore always be presented alongside the raw Bayes factor values, such as in Fig. 10.

## Discussion

As the saying goes, “there are as many opinions as there are experts” (Roosevelt, 1942). In Bayesian inference, these differences in opinion can become particularly important in the context of prior elicitation from experts. Here, we investigated how the interpersonal variability of elicited prior distributions influences the results of Bayesian null hypothesis testing on the basis of a large database of psychological studies. We introduced three different sensitivity analyses and concluded that the qualitative conclusions of Bayesian hypothesis tests are rarely affected by the prior distributions, but that the quantitative results can differ substantially.

The sensitivity of the Bayes factor has often been a subject of discussion in previous research (e.g., Berger, 1990; Sinharay and Stern, 2002). However, to our knowledge, our paper is the first one to provide a structured analysis of the sensitivity of the Bayes factor in the light of prior distributions that were elicited from psychology experts. Our results give an impression of the extent of interpersonal variability between elicited prior distributions that can be expected in psychological research, and we show that the Bayes factor is sensitive to this variability. However, our results also demonstrate that the use of different elicited prior distributions does not necessarily change the direction of the Bayes factor or the category of evidence strength. In fact, for our elicited priors, the majority of qualitative test conclusions remained unaffected by the priors. This insight may increase the support for informed Bayesian inference among researchers who were worried that incidental fluctuations in expert opinions might determine the qualitative outcomes of their Bayesian hypothesis tests. However, as we argue below, it should not be taken as evidence that informed prior distributions will generally not affect test decisions. This depends on the models being compared, the available data, and the degree of information of elicited priors.

Beyond displaying the consequences of interpersonal variability in prior elicitation, our analyses can also be used as a guidance for future Bayes factor sensitivity analyses. Our paper demonstrates how prior elicitation can be used to identify relevant prior distributions, and provides a structured approach for the succeeding sensitivity analyses. By analyzing the direction and evidence category of the Bayes factor, researchers can investigate whether their candidate prior distributions affect the qualitative conclusions of their Bayesian hypothesis test. Additionally, researchers can investigate the quantitative differences between the Bayes factors using the different prior distributions. As we demonstrated in this paper, the proposed approach allows researchers to infer from sensitivity analyses that the Bayes factor is at the same time robust and sensitive to the choice of the prior distribution. Specifically, qualitative conclusions based on the Bayes factors can be highly robust against the choice of the prior distribution, while the absolute value of the Bayes factor is sensitive to the prior distribution.

It is interesting to note that elicited prior distributions do not always lead to higher Bayes factors than default prior distributions, even though they display less uncertainty about parameters. Our results show that there are two keys to understanding the relationship between informed prior distributions and Bayes factors. First, to yield higher Bayes factors, informed prior distributions need to increase the discriminability between the models. If the informed prior distribution mimics the point prior under the null model (as was the case for Expert 1), the discriminability between the models is low, which leads to a relatively low strength of evidence. Second, the predictive accuracy of informed prior distributions is rewarded. Specifically, Bayes factors are highest if the effect size in the sample falls within the range of parameter values that were predicted by the informed prior. We argue that understanding these relationships is not only crucial for the interpretation of sensitivity analyses, but can also be important for Bayesian design planning, where researchers determine the sample size of studies based on the prospective strength of evidence (Stefan et al., 2019). Typically, larger sample sizes are needed to obtain strong evidence if the compared models are less discriminable, and smaller sample sizes are required with informed models where one of the models makes accurate predictions. Of course, this should not lead researchers to aim solely for design efficiency. It remains important that the statistical models reflect theoretical beliefs and make realistic predictions. Therefore, prior specification should always precede sample size planning in practice.

The variability of prior distributions and their impact on the results of Bayesian hypothesis tests immediately raise the question whether one prior distribution can be considered superior to another. Following de Finetti’s subjective notion of probability (de Finetti, 1974), prior distributions can neither be discussed nor critiqued as they represent the idiosyncratic belief of an individual. An independent researcher who elicited prior distributions from multiple experts would therefore have no reason to prefer any elicited prior over another. However, even though a single prior distribution cannot be evaluated from a normative standpoint, it can be evaluated regarding its concordance with other elicited prior distributions. For example, in our study, Expert 1’s priors deviated substantially from all other experts. This does not necessarily mean that Expert 1’s prior distribution is any less valid than the other experts’ priors. However, the divergence can instigate further investigations into reasons for the apparent disagreement. Possible reasons include that the expert holds minority beliefs or possesses different information from the other experts, but also that the expert misunderstood the elicitation procedure or did not participate faithfully. In practice, it might be necessary to contact the expert again after the elicitation to obtain this information. Another way to compare prior distributions is by means of their predictive accuracy in the light of data. This can be achieved by computing Bayes factors between models using different elicited priors, as was done in the previous section of this paper in the context of a sensitivity analysis (cf. Fig. 12). As we argue below, this approach should never be used to cherry-pick priors after the results are known. It can, however, be used to select experts for future elicitations, or to compute knowledge-based weights for the aggregation of future elicited priors from the same group of experts (Wilson & Farrow, 2018).

Even though prior distributions can exert considerable influence on the Bayes factor value, it is important to note that priors should not be chosen solely because of their influence on the Bayes factor. Researchers might be tempted to choose a convenient informed prior after the data are known to increase the evidence obtained from the data. For example, a devious researcher might choose a prior distribution that peaks on unrealistically high effect sizes or a prior that is exceedingly wide to obtain spurious evidence in favor of the null model, or define “oracle priors” (Dienes, 2008), that is, point priors on the maximum likelihood estimate in the data, that distort evidence in favor of the alternative model. These prior specifications no longer represent valid pre-data theoretical assumptions, and thus prohibit severe tests of theory (Mayo, 1991). We wish to stress that prior distributions are subject to public critique; researchers who cherry-pick prior distributions with the sole purpose of skewing the results in their favor will struggle to defend these prior distributions in the (post)peer-review process. Ultimately, prior distributions are part of the model specification and subject to the same scrutiny as, say, the selection of a likelihood function. To avoid the suspicion of post-hoc theorizing, it is recommendable that researchers specify the prior distributions before the data collection, and record their decisions in a preregistration (Crüwell & Evans, 2019; Stefan et al., 2020; Chambers, 2013). A prior sensitivity analysis, as presented in this paper, can go hand in hand with the preregistration and further increase the transparency of a study. Similar to a multiverse analysis (Steegen et al., 2016), computing analysis results for different elicited prior distributions can bring subjective decisions in the statistical analysis to light and make researcher degrees of freedom transparent. Thus, prior sensitivity analyses can provide researchers with interesting information about the robustness of their results and can increase their confidence in their conclusions. It is important to note though that the prior distributions included in a sensitivity analysis should all be justifiable for the specific research context at hand. Prior distributions elicited from field experts for a well-defined research question typically fulfill this criterion.

Like all other measurement methods, prior elicitation is subject to measurement error (O’Hagan, 2019; Stefan et al., 2020). Therefore, differences between experts can both be a result of their different theoretical convictions of the experts and measurement fluctuations. To date, little research has been conducted to assess the amount of measurement error in prior elicitation. In our study, we decided to use one of the most common prior elicitation methods (Morris et al., 2014), and gave experts the opportunity to adjust the elicited prior distributions. It is important to be aware that these methodological decisions in the prior elicitation procedure might have influenced the elicited prior distributions (Stefan et al., 2020). However, our results indicate that small differences in elicited prior distributions barely play a role in Bayesian inference. Therefore, Bayes factors can be considered robust against small measurement inaccuracies in the prior elicitation process. However, they are not robust to large, potentially systematic biases. This emphasizes the importance of well-validated prior elicitation methods that minimize potential cognitive biases (O’Hagan, 2019; Kahneman, 2011; Tversky & Kahneman, 1983). It is beyond the scope of the current study to investigate the validity of different prior elicitation methods, but we believe that this can be a valuable avenue for further research.

The prior elicitation effort reported in this paper is special in several ways. Rather than conducting a prior elicitation for a specific effect or research design, we asked experts to provide their assessments for generic small-to-medium effect sizes in their field that are larger than zero. This allowed us to include experts from different research fields and establish a minimum level of consent between the participating experts. However, it also means that the elicited prior distributions are influenced less by substantive theory than they may be in a typical prior elicitation context. Moreover, the lack of experimental context means that experts’ beliefs were unrestricted by any particular operationalization. It is possible that experts would display more certainty and less disagreement if prior distributions were elicited for a specific psychological effect or for a particular research design. Another noteworthy aspect of our elicitation effort is that we elicited beliefs for standardized effect size coefficients, rather than, for example, raw differences in group means. Of course, this is partly due to the fact that we did not refer to a specific experimental context. However, we believe that eliciting beliefs about standardized parameters generally has several advantages. Since individual studies and meta-analyses mostly report standardized effect sizes, it will arguably be easier for experts to include this knowledge into their priors. Additionally, standardized parameters might steer the experts’ focus towards general theory and scientific evidence, rather than intuitions about a particular experimental context. Thus, prior distributions elicited for standardized parameters might be more connected with theory and less influenced by measurement tools. However, the influence of standardization on prior elicitation results is still an open empirical question.

The results in this paper are subject to several limitations. First, all results of the sensitivity analyses are dependent on the databases and statistical tests that were used. We carefully selected the databases to be representative for psychological research and the two hypothesis tests we investigated are among the most frequently used tests in psychology (Wetzels et al., 2011; Bosco et al., 2015). However, different dataset compendia or hypothesis tests might yield different levels of Bayes factor sensitivity. Therefore, the effects of interpersonal variability in prior distributions demonstrated in this article should always be interpreted in the context of the current application scenario. Second, we only elicited prior distributions from six experts. Although this number of experts is within the recommended range for domain-specific prior elicitation efforts (O’Hagan, 2019; Grigore et al., 2013)^6^ and can be considered a realistic sample size for practical applications, it is possible that more variability would have been observed if more experts had participated in the elicitation effort. Future studies could therefore extend our analyses to more experts, different research questions, and statistical models. In this context, it should also be stressed that despite the generality of our elicitation question, the idiosyncratic prior distributions of six experts from a single university should not be mechanically applied as universal “informed default” priors for psychological science. In our opinion, establishing such “informed default” priors for a well-defined research field is possible, but requires a broader empirical base (for an example, see McKinney et al.,, 2021). Third, our paper focuses solely on Bayes factors. Although Bayes factors are frequently used in practice (van Doorn et al., 2019), some experts prefer other Bayesian model evaluation methods or focus on posterior inference (Vehtari et al., 2017; Kruschke, 2011; Evans, 2019; Gelman et al., 1996). These alternative methods are also influenced by the prior distributions on parameters. It would therefore be interesting to investigate the influence of differences in elicited prior distributions on these methods as well.

The fact that the results of a statistical analyses depend on the statistical models, has long been known as ‘Jeffreys’s platitude’ (Jeffreys, 1961). By including different knowledge about prior parameters in Bayesian model comparisons, researchers change the involved models, and therefore pose different statistical questions that prompt different statistical answers. It is therefore not a weakness, but a strength of Bayes factors to be sensitive to the specification of the prior distribution. Here we demonstrated that the extent to which the statistical answer differs, depends on the differences in the questions asked. Modest differences in elicited expert knowledge are still visible in the statistical results, but rarely change the qualitative conclusions of the model comparison. Concerns that idiosyncrasies between experts might jeopardize the objectivity of their statistical analyses are easily overstated. We hope that this insight will lead more researchers to embrace informed Bayesian inference with elicited prior distributions in the future.

## Open Practices Statement

Associated materials can be found at https://osf.io/vqszj/. Reproducible analysis code is available at https://osf.io/vqszj/ and in the connected GitHub repository (astefan1/ ExpertAgreement). The two study databases used in this article are openly accessible, but cannot be shared on the OSF repository accompanying this article due to licensing issues. However, instructions for downloading and cleaning the data can be found on the authors’ OSF and GitHub repository. All data from the prior elicitation effort reported in this article is available on the OSF repository (https://osf.io/vqszj/). Reported analyses were not preregistered.

## Footnotes

1 But note that the Bayes factor allows you to compare any models you can specify (Etz et al., 2018; Evans & Servant, 2020). If there is no analytic solution for solving the integrals, Bayes factors can be approximated using numerical methods (e.g., Gronau et al.,, 2017; Evans & Annis, 2019).

2 For corresponding figures of raw Bayes factor values, see our Online Appendix https://osf.io/vqszj/.

3 Correlation coefficients in the Bosco et al., (2015) database are reported with three decimal places. For Fig. 11, we used correlation coefficients with one significant decimal place (e.g., *r* = − 0.200).

4 In the original study context, a negative effect size may in fact be consistent with the pertinent alternative model. However, for the sake of demonstration we assume that the alternative model is defined by the elicited positive-directional prior distributions: this allows us to examine a broad range of outcomes, both consistent and inconsistent with the direction of the hypothesis.

5 A corresponding figure for the Bosco et al., (2015) dataset can be found in our Online Appendix on https://osf.io/vqszj/

6 Note that these recommendations are based on experiences of individuals with lots of experience as facilitators of prior elicitation efforts, but lack a broader empirical basis.
